# There’s no time for no stress! Exploring the relationship between pharmacy student stress and time use

**DOI:** 10.1186/s12909-023-04266-5

**Published:** 2023-04-24

**Authors:** Lana M. Minshew, Hannah P. Bensky, Jacqueline M. Zeeman

**Affiliations:** 1 Robert D. and Patricia E. Kern Institute for the Transformation of Medical Education and the Department of Clinical Sciences in the School of Pharmacy, Medical College of Wisconsin, Milwaukee, WI USA; 2 Eshelman School of Pharmacy, University of North Carolina at Chapel Hill, Chapel Hill, NC USA; 3 Division of Practice Advancement and Clinical Education, Eshelman School of Pharmacy, University of North Carolina at Chapel Hill, Chapel Hill, NC USA

**Keywords:** Stress, Time, Well-being, Mixed methods, Challenge-hindrance stressor framework

## Abstract

**Background:**

Health professions students experience significantly more stress than students 20 years ago. While prior studies have explored student time use and other studies have begun to explore factors influencing student stress, little is known about the relationship between student time use and stress. As more efforts are employed to promote student wellness and better understand student stress, it is imperative to recognize the implications of time as a finite resource. Thus, it is important to understand if and how time use relates to student stress so the two can be better managed.

**Methods:**

A mixed methods approach exploring the challenge-hindrance stressor framework was used to collect and analyze student stress and time use. First, second, and third year pharmacy students were invited to participate. Participants completed the Perceived Stress Scale (PSS10), a week-long daily time logging activity, and daily stress questionnaire. After the week-long daily time logging, students participated in a semi-structured focus group. Descriptive statistics were used to analyze quantitative data, and inductive coding along with creation of summary reports were created for the qualitative data.

**Results:**

Students reported moderate stress on the PSS10, and spending the majority of their time on activities of daily life and academic activities. Students shared that academics, co-curriculars, and working for pay increased their stress, whereas discretionary activities such as socializing and exercising alleviated stress. Finally, students reported feeling overwhelmed due to too little time to complete all necessary activities daily, including limited time to invest in discretionary activities to support their wellbeing.

**Conclusions:**

Increased stress levels among students is a concerning trend that affects students’ mental health and therefore limits their ability to perform to their greatest potential. Better understanding of the relationship between time use and stress is critical to improve the quality of life of students in the health professions. These findings provide critical insight into factors contributing towards student stress that can inform curricular strategies that support wellness within health professions education.

**Supplementary Information:**

The online version contains supplementary material available at 10.1186/s12909-023-04266-5.

## Background

Stress among health professions students (HPS) is a well-documented phenomenon [1–5] with students from all health professions experiencing a multitude of academic and personal stressors [5, 6]. Stress is found to impact mood, as well as increase instances of mental disorders and suicide risk, and this risk is greater in HPS [7]. Instances of stress begin through the competitive application process and continue throughout the healthcare education process as the need to excel in courses, exams, and other high stakes situations are constant [7].

Research has shown that HPS experience significantly more stress than HPS did 20 years ago [5]. This is reflected in students’ health-related quality of life scores meeting the threshold for risk of major depression or dysthymia [2, 8]. Specifically, pharmacy students were found to have diminished mental health and significantly increased stress during the pre-clinical years [3]. Student perceived stress is identified as the strongest predictor of emotional exhaustion and lack of accomplishment [9] and higher stress levels can be linked to increased expectations regarding academic performance [6, 10]. For instance, one study found that students felt guilty for not using free time for academic work, leading to little time spent on leisure activities to support a balanced lifestyle [10].

The challenge-hindrance stressor framework, initially created in management research, has recently been implemented in higher education. Stressors are categorized into two categories: challenge stressors, which prompt normal to moderate psychological stress that positively impact performance, and hindrance stressors, which cause distress and negatively impact performance [11–14]. The distinction of a stressor as a challenge or hindrance is appraised by the person and thus can vary across individuals [12]. The distinction between challenge and hindrance stressors is the appraisal of the stressor as an opportunity to grow in an area related to the tasks that must be accomplished [12]. The framework acknowledges that all stress is not necessarily undesirable, but can actually serve as source for motivation, and that this distinction is dependent upon individual discretion [11].

Travis and colleagues explored the utility of the framework in higher education to explore stress and academic outcomes [11]. Their findings were theory consistent and support the use of the challenge-hindrance stressor framework in educational contexts. Advancing this work, the application of the challenge-hindrance stressor framework to explore HPS stress and time use beyond academics (e.g., academic outcomes) remains unstudied.

While there is a plethora of studies that evaluate HPS stress, little is known about how student time use relates to stress. When discussing stress, medical and pharmacy students identify lack of time to oneself and an inability to balance school [15] and personal responsibilities as stressors [16]. Previous research exploring pharmacy student time use has focused on academics, particularly time spent on academic activities and its correlation to student achievement [17, 18]. However, time used for academic purposes is only one component of students’ time and may not represent other stressors that influence student outcomes [19]. Zeeman and colleagues conducted a time use exercise exploring pharmacy student time use beyond just academic time; however, the study did not explore its relationship to student stress [19].

As more efforts are employed to promote HPS wellness and better understand student stress, it is imperative to recognize the implications of time as a finite resource. Thus, it is important to understand if and how time use relates to student stress so the two can be better managed. While studies have examined both HPS stress and time use, a gap in the research exists regarding how the two relate. Further, the majority of existing literature utilized surveys to investigate stress and student time use. While this methodological approach can facilitate large sample sizes, it creates an incomplete picture of all that impacts student time use and stress and limits the narrative quality of the participant experience. For instance, it is difficult to capture duration of time spent on task as well as gather in-depth insight into participant perspective on the interplay of time spent on activities and activities that cause or alleviate stress with a single administration survey.

Using a mixed methods approach, the purpose of this study was to explore the relationship between pharmacy student stress and time use and the interaction between the two constructs. The challenge-hindrance framework provided a lens for which to analyze student perceptions regarding all activities that cause stress in their lives.

## Methods

### Data collection

**Fig. 1:**
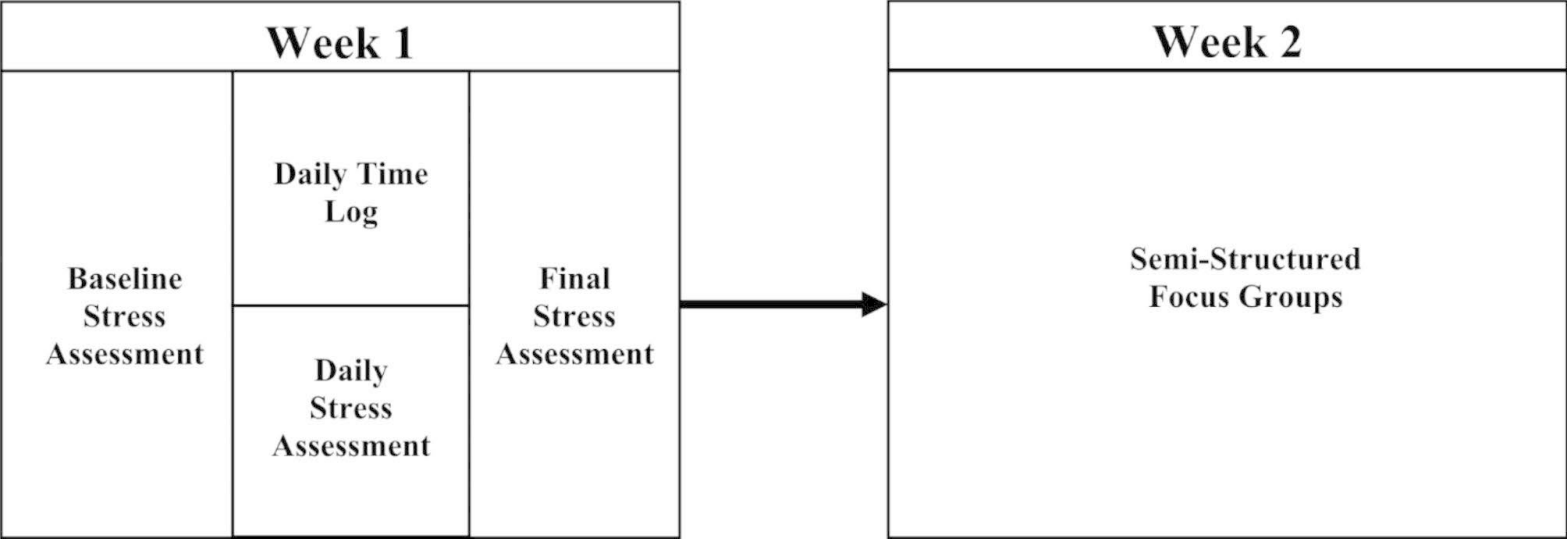
Mixed Methods Study Design Exploring Student Stress and Time Use

This study utilized an observational, mixed methods approach involving stress questionnaires, daily time logging, and semi-structured focus groups to explore the relationship between pharmacy student time use and stress (Fig. 1). All students in the first, second, and third (PY1-PY3) years of a Doctor of Pharmacy (PharmD) program completing didactic course work in August – September 2020 (n = 362) were eligible to participate in the study. Students on clinical rotations were excluded from the study, as it was hypothesized that student time use and stress would differ between these educational settings. Students were recruited through in-class presentations during didactic courses. Data collection occurred in the Fall 2020 semester, during the ongoing COVID-19 pandemic. While not a primary study objective, this context is important, as the developing pandemic was an added stressor participants discussed despite not being probed on the topic.

A sample of 16 student volunteers were recruited for this exploratory study. The time-logging weeks were selected based on the week being representative of an average week in terms of workload for courses for each year in the program and occurred approximately halfway through each cohort’s semester. For instance, the weeks selected did not have major exams or objective structured clinical examinations (OSCEs) scheduled.

One week prior to the time log week, students completed a baseline stress assessment using a modified Perceived Stress Scale (PSS10) survey via online survey to measure stress levels [20]. Prior studies have demonstrated PSS10 reliability when measured between two day and four weeks intervals; [21] thus, the original PSS10 was modified to assess student stress during the last week as opposed to during the last month in this study. The baseline PSS10 survey included 10 questions regarding common feelings of stress, including feeling upset, out of control, irritated, and overwhelmed [20]. Participants completed daily time logging for one-week, tracking their time via a developed Excel template each day in 30-minute intervals. All participants were provided training materials and example scenarios that included the ten predefined time use categories to provide a more consistent method of capturing student time use (Table 1) [19]. For instance, students were instructed that conducting activities of daily living (ADL) was inclusive of time spent conducting activities such as hygiene, dress, meals, childcare, grocery shopping, laundry, etc. and participating in co-curriculars was inclusive of time spent participating in student organizations, service/outreach opportunities, career/professional development, etc.

**Table 1:** Weekday and Weekend-day Student Time Use^a^

	Weekday Hours N = 16 Mean (SD)	Weekday Hours N = 16 Range	Weekend-Day Hours N = 16 Mean (SD)	Weekend-Day Hours N = 16 Range
**Activities of Daily Life**	10.6(2.5)		11.7(2.7)	
Sleeping	7.8(1.3)	4.5–11.5	8.7(1.4)	6.0-11.5
Commute to School^b^	0.0(0.1)	0–1.0	0.2(0.6)	0–2.0
Conducting other ADL	2.8(1.3)	0.5–7.5	2.8(1.1)	0.5-6.0
**Academic Activities**	9.0(3.4)		4.8(3.0)	
Attending Class	3.7(2.1)	0-9.5	0(0)	0–0
Studying or Coursework	3.5(2.3)	0-9.5	4.1(2.8)	0-12.5
Participating in Co-curriculars	1.8(2.3)	0–10.0	0.7(1.3)	0-4.5
**Discretionary Activities**	4.3(2.8)		7.4(3.8)	
Exercising	0.4(0.5)	0–2.0	0.5(0.8)	0–3.0
Engaging in Social Activities	1.6(2.2)	0–12.0	2.2(2.6)	0-9.5
Viewing Media or Social Media	1.8(1.7)	0–8.0	1.8(1.5)	0-5.5
Working for Pay	0.5(1.3)	0–5.0	2.9(4.2)	0-11.5

^a^ Time data included in calculations if ≥90% of daily time log completed; mean hours may not equate to 24.0 h

^b^ All classes during study period were conducted virtually due to the COVID-19 pandemic. The school building was open and available for student use should they need a location to connect to online classes and/or study as long as they followed the COVID-19 protocols in place at the time.

In conjunction with logging their daily time use, participants completed three questions evaluating their stress daily. The first question required participants to identify how often they felt stressed during the day using the same scale as the PSS10. The other two questions asked participants to provide 1–3 examples of things that caused stress and alleviated their stress that day.

At the conclusion of the time logging week, all students completed the modified PSS10 survey and participated in a 60-minute semi-structured focus group via Zoom. Four focus groups were led by two non-faculty members of the research team to create an open and free-exchange dialogue among participants about their experiences. Students were allowed to turn their camera on or off and change their screen name as desired to protect their identity. The study was approved by the institutional review board (IRB # 20–0872).

### Data analysis

The 10 predetermined time use categories from the time logging instrument were organized into three overarching categories: (1) activities required for daily life, (2) academic activities directly related to school, and (3) discretionary activities chosen by students (Table 1) [19]. These categorizations were intentionally selected to explore time spent on activities necessary for human life (e.g., sleeping, eating, hygiene), activities that can be influenced by the academic institution (e.g., classes, coursework, co-curriculars), and all other activities participants may choose to engage in during their discretionary time that may vary from person to person (e.g., exercising, socializing, working for pay). Time log data were excluded from analysis if the student failed to complete at least 90% of time logging for that day. Descriptive statistics were used to analyze time use data by weekdays and weekend-days, as prior studies noted that student time use varied during these periods [19].

The modified PSS10 baseline and final stress assessments taken by participants were scored and analyzed according to PSS10 guidelines on the 0–40 point scale: 0–13 = low stress, 14–26 = moderate stress, 27–40 = high stress [20]. Both PSS10 assessments required participants to respond to each of the 10 items prior to submitting their stress assessment; thus, there could be no missing responses within the questionnaire upon submission. Baseline and final stress assessments were compared using paired t-test and descriptive statistics were used to analyze Likert items on the daily stress assessment questionnaire (Table 2). An iterative process of deductive and inductive coding was used to analyze the two open response questions on daily causes of stress and what students did to alleviate their stress. Microsoft Excel Version 16.16.17 was used to conduct all analyses. Missing data were removed prior to analysis of the completed study components.

**Table 2:** Students’ Reported Daily Stress

Student reported daily stress^a^	Weekday (n = 16) N (%)	Weekend-day (n = 16) N (%)
Often	36 (45%)	4 (14%)
Sometimes	32 (40%)	14 (48%)
Never	12 (15%)	11 (38%)

^a^Daily stress questionnaire evaluating “How often did you feel stressed today?” (0) Never, 1 (Almost Never), 2 (Sometimes), 3 (Fairly Often), 4 (Very Often). Data summarized as Often (4-Very Often, 3-Fairly Often), Sometimes (2-Sometimes), and Never (1-Almost Never, 0-Never).

Focus group data were recorded and transcribed by Zoom. A single research team member inductively coded a single focus group to create an initial codebook. The other team members used the developed codes to independently code the same focus group transcript. The team then discussed the developed codes, any newly identified codes and their application, and any discrepancies until consensus was achieved. The same process was followed for the remaining focus groups. Inter-coder agreement for the entire data set was found to be above 80%, the accepted threshold for qualitative data [22]. Constant comparison method for theme generation was utilized to develop summary documents to highlight the most commonly applied codes.

## Results

Sixteen students participated in the study across the three didactic years of the program: three PY1, two PY2, and 11 PY3 students. The total sample size was deemed sufficient given the exploratory nature of this study. Data are reported as a single group and does not distinguish between program year considering the cohort-based sample sizes. Across all participants, 97% (n = 263/272) of the study activities (i.e., baseline PSS10, daily time log, daily stress questionnaire, final PSS10, focus group) were completed, resulting in 3% of study components to be missing (i.e., 2 daily time logs from 2 students, 7 daily stress questionnaires from 7 students). All participants completed both baseline and final PSS10 as well as participated in a focus group.

Students spent the majority of their time on activities of daily life (ADL) during both weekdays (44%) and weekend-days (49%), spending a mean 10.6 h/weekday and 11.7 h/weekend-day on ADL (Table 1). For academic activities directly related to school, students reported spending on average 9.0 h/weekday attending class, studying, completing coursework, and/or participating in co-curriculars, accounting for 37% of their weekday time use. Their average time spent on these activities reduced over the weekend to 20% of their weekend time use with students reporting spending 4.8 h/weekend-day on studying, completing coursework and/or participating co-curriculars. Time spent engaging in discretionary activities such as exercising, engaging in social activities, viewing media, and working for pay were lower, accounting for 18% (4.3 h) of weekday time use and 31% (7.4 h) of weekend time use (Table 1).

Overall, students reported moderate stress on the baseline and final PSS10 questionnaire. While, students scored higher on the final PSS10 stress questionnaire (mean: 18.3, SD: 5.58) than on the baseline PSS10 assessment (mean: 17.8, SD: 6.57), the difference was not statistically significant (t(30) = -0.20, p = 0.84). For the daily stress question, students mainly reported feeling stressed “Sometimes” (40% of responses) and “Fairly Often” (31% of responses) on weekdays, whereas they reported feeling stress “Sometimes” (48% of responses) and “Almost Never” (31% of responses) on weekend-days (Table 2).

Students indicated their main daily causes of stress were related to academic time use (e.g., exams, quizzes, class assignments, grades), having too little time to complete tasks, and anticipatory stress for future events (e.g., exams, quizzes). A student wrote they had, “concerns about grades (midterm exam, quiz), feelings of being overwhelmed by busy or loaded weeks.” Another student shared they were stressed due to “studying for exams, working on assignments, and thinking about how stressful this week will be.” Physical activity (e.g., exercising, running), activities of daily living (e.g., napping, eating), social time, interacting with media (e.g., Netflix, social media platforms), personal hobbies (e.g., baking, watching football), and treating oneself to a reward (e.g., takeout, facial) were all ways students reported coping with daily stress. Students listed “playing with my cat, baking brownies after the exam, watching Brother Bear with my roommate over a bottle of wine,” and “took a nap, got iced coffee, took a (masked) walk with friends” as strategies they used to cope with daily stress.

Focus group themes identified commonalities across students, identifying time use activities associated with increased and decreased stress, as well as coping strategies (Table 3). Students noted academics, co-curriculars, and time spent working for pay increased their stress. One student stated, “my stress is dependent on the school stuff we have going on.” Whereas another student felt their co-curricular involvement was the most stressful, saying “co-curriculars, I would say that is a huge part of my stress.” Working for pay was also noted to increase stress, with one student sharing, “I would say it [work] is stressful … yeah, it is a big part of my stress.” Further, students also reported feeling anticipatory stress knowing they had coursework to complete and tests to study for. For instance, one student shared, “it’s just like thinking about the list of assignments or whatever I have to do…is what really seems to stress me out.”

**Table 3:** Most Commonly Applied Codes to Focus Group Discussion

Academic Time	Co-Curricular Time	Work for Pay	Social Time	Effective Coping	Ineffective Coping
1. Increased Stress	1. Increased Stress	1. Increased Stress	1. Decreased Stress	1. Decreased Stress	1. Increased Stress
2. Decreased Stress	2. COVID-19	2. Decreased Stress	2. Increased Stress	2. Physical Activity	2. Prioritization
3. Virtual Learning	3. Decreased Stress	3. Too Little Time	3. COVID-19	3. Personal Hobbies	3. Interaction with Media
4. COVID-19	4. Prioritization	4. Guilt Saying No	4. Contributing Personality Trait	4. Lack of Effective Stress Relief	4. Too Little Time

While the majority of students indicated academics, co-curriculars, and time spent working for pay increased their stress, a handful of students (n = 5) shared a different perspective. For example, one student shared, “I feel like the more time I spend studying the less stress I feel” and another stated, “…attending class and learning doesn’t stress me out…I feel really good about myself for being in class and being focused.” When it came to co-curriculars, a student stated, “…actually attending those [co-curricular] meetings I end up enjoying it more and it’s nice to see everybody.” Finally, a student shared, “It’s [working for pay] a big part of my stress, but the financial aspect alleviates stress for me too because not having any income for three years would stress me out big time.”

Time reserved for social activities, overall, was a source of decreased stress with a student saying, “the more I socialize, the less stressed I am” and another student shared, “I don’t think I realized how much [socializing] helped me until this COVID stuff … I took it for granted before and didn’t realize how much it helped me.” However, some students reported social activities as a source of increased stress because it took time away from schoolwork. One student shared, “With social time I try to take breaks and its fun in the moment, but I also feel like I have this guilt sometimes for taking that time…and it just feels like I’m procrastinating, which then becomes a stressor instead of a stress relief.” To ensure they were making time to socialize, a student noted, “I find myself scheduling my social time into my Outlook calendar and then it pops up on my phone and I’m like ‘Oh great. Now I have to do this’ and it becomes a chore instead of something that’s fun, which adds to my stress.”

Students associated increased stress with feelings of having too little time to complete all necessary tasks in a single day. Limited time led to increased stress levels and prevented students from participating in activities that would decrease their stress, like exercising, taking a break, or spending time with family and friends. One student shared, “I want to do things to de-stress myself but also if I’m doing stuff to de-stress myself, then I’m not doing the thing [school] that’s stressing me out.” Another student echoed this sentiment by saying, “I think that it’s hard to not be stressed out when you’re hanging out with your friends and you feel like you need to do other things.” Feeling as though there is too little time encouraged students to be strategic in how they spent their time. For instance, a student shared, “I’m big on calling my family while I’m making dinner because then I’m being productive and not wasting time on the phone with my mom for hours when I should be doing other things.” In addition to too little time, students also reported feeling as though they did not have effective methods to cope with stress. One student stated, “I need to be more proactive with how I manage my stress, I sometimes tend to be reactive and just impulsively do something like go take a nap or get a milkshake,” and another student shared, “when I get overwhelmed and stressed out I don’t have a good coping method and that was really highlighted that week [time logging week].”

Students described how the ongoing COVID-19 pandemic impacted their stress levels regarding academics, co-curriculars, and how they socialized. For instance, students indicated that virtual learning made it difficult to take breaks from school. One participant shared, “the virtual environment has made it very easy to sit at your desk all day long and only do school.” A student in a leadership position for a co-curricular acknowledged that “COVID … is an added burden [because] now I have to change all of [the plans] and work under completely different circumstances which has definitely added to my stress.” Students also noted that COVID-19 made it more difficult for them to participate in stress relieving activities, like exercising and socializing. For example a student shared, “because of COVID I’m not actually going to the gym … so that limits the activities I can do” and another noted, “with COVID it’s just harder to see people.”

## Discussion

This study is one of the first to explore the relationship between student time use and stress using mixed methods and the challenge-hindrance framework. Students spent the majority of their time on activities of daily living and academic activities related to school, leaving little time for discretionary activities of their choice and activities effective for coping with stress. These findings provide critical insight into the relationship between student stress and time use, and can assist health professions educators with optimizing academic expectations and their associated time requirements. Recognizing time as a finite resource, it is important to ensure pharmacy students and all HPS have sufficient time to complete daily functional tasks (e.g., sleeping, eating) in addition to academic requirements while supporting students with appropriate resources (e.g., time, strategies) to effectively cope with stress and promote sustained wellness.

HPS consistently report increased stress due to academic performance [23, 24] and our participants were no different. Students reported moderate stress levels on the baseline and final assessments, and often feeling stressed during the weekdays. Students shared that academic deliverables, having too little time to complete required work, and anticipatory stress related to upcoming responsibilities increased their daily stress, whereas taking a break, spending time with friends or pets, and physical activity helped to alleviate stress. In focus groups, students reported increased stress associated with academics, co-curriculars, and time spent working for pay; whereas decreased stress was associated with time spent on social activities, physical activity, and personal hobbies. The COVID-19 pandemic and online learning was a major discussion point of students in the focus groups, despite this not being a focus of the study.

Student assessment of stress aligns with the challenge-hindrance stressor framework in that individuals who appraise a stressor as an opportunity for growth (i.e., challenge stressor) feel pushed and energized to rise to the challenge [11, 12]. Whereas when an activity was assessed as depleting (i.e., hindrance stressor), it increased student stress. Further the individual appraisal of stressors varied across participants when it came to classifying certain activities as a challenge or hindrance stressor and ultimately influenced their ability to cope with stress. For instance, some students identified attending co-curricular events and class sessions as stress relieving activities, in that despite being difficult and time consuming, they felt good when participating in the activity (i.e., challenge stressors). While the majority of students did not report this sentiment, this observation may support Alshammari’s findings that there is not a significant relationship between academic stress leading to poorer academic performance [1]. Whereas many students found school related activities to be sources of stress and viewed as overwhelming (i.e., hindrance stressors). Similar to McCauley and Hinojosa’s [12] findings, activities could transition from decreasing student stress to becoming a hindrance stressor due to the individual re-assessing the activity. For example, hanging out with friends could transition from a stress relieving activity to being viewed as procrastination, thus becoming a hindrance stressor for students.

Related to academic performance is the guilt students expressed feeling if they engaged in leisure activities instead of engaging in academic endeavors. Leisure activities, such as spending time with friends and family, could both decrease and increase their stress. Some even equated leisure time with procrastination, thus increasing their stress instead of alleviating their stress. This is concerning as leisure activities facilitate recovery from academic and other hinderance stressors. Further, it’s been noted that social activities help to reduce stress in medical students [10]. Students need an outlet and effective coping mechanism from their academic programs in order to reduce the risk of burnout and depression while also promoting a healthy lifestyle [2, 10, 25, 26]. For instance, Lemay et al. found that introducing students to yoga, meditation, and other mindfulness activities decreased student stress [27].

There are only 24 hours in a day and when students are spending nearly 20 hours on essential activities for daily living and tasks necessary for their professional degree, minimal time remains for them to engage in discretionary activities, including those that are effective for managing stress. Pharmacy students in this study and previous research [28] identified lack of time to complete all tasks in a day and their inability to balance school and other life activities as stressors. McCauley and Hinojosa suggest that when students believe they have the appropriate resources to respond to stressors, those stressors are more apt to be appraised as challenges rather than a hindrance [12]. One way is to provide students with the necessary cognitive tools to reappraise and reframe their mindset regarding challenge stressors. Studies show that students who are provided with reappraisal or reframing stress techniques outperform control groups on the Graduate Record Examination (GRE) [29] and report lower stress on self-report perceived stress scales [30, 31].

Other support needs to be from leadership and the organization; thus when promoting student wellness, it is imperative that health professions curriculum designers and faculty are aware of the time it takes to engage with and complete curricular requirements on a weekly, monthly, and semesterly basis. One strategy our institution employs is the use of a master assessment calendar. This calendar includes all major and minor assessments for each cohort, and is reviewed prior to the start of each semester to (1) optimize assessment load distribution across the term, and (2) promote course director awareness of curriculum requirements students are engaged in outside of their direct class. Further, this calendar is reviewed closely to better support our students and target wellness initiatives to times of need.

Additionally, the assessment calendar has facilitated faculty discussions regarding assessment load and opportunities for optimization, as students reported the volume of academic deliverables and feelings of having too little time to complete all required tasks as factors contributing towards their stress. Bergmann and colleagues found that students feel guilty for not using free time for academic work, [10] a sentiment shared by participants in this study. This can result in less time spent on stress alleviating activities, which in turn can further perpetuate student stress and decrease student wellness. An additional strategy our institution has used to combat this is optimizing academic assessment load where appropriate. For example, one course consolidated twice weekly assignment deadlines to once weekly. The master assessment calendar assists with this process as academic deliverables and deadlines are reviewed in totality across the curriculum, rather than in isolation by each course.

The study period was intentionally selected as a representative week of a typical workload of the semester, one that did not include any high stress events such as major exams or Objective Structured Clinical Examinations (OSCEs), as these events are known stress triggers for students [10, 23, 24]. The non-statistical difference observed between the baseline and final PSS10 assessments supports this as we anticipated stress levels to remain fairly constant during the study period. Thus, our findings may only reflect this timepoint and not be reflective of mid or late semester stress students experience (e.g., midterms, final exams).

Although this study was designed prior to the COVID-19 pandemic, all data were collected during the pandemic at a time when students were engaging in academic activities remotely, social distancing was highly encouraged, and vaccines were not readily available. These circumstances influenced our study’s findings as students noted how their transition to online learning influenced how they spent their day. Students acknowledged that continuously sitting at their computers made it easier to constantly engage in academic work, while neglecting other aspects of their life, such as social interaction and physical health. Particularly, students discussed the negative impact COVID-19 had on their ability to spend time with friends and family, aligning with Hagemeier & Dowling-McClay’s findings that students have experienced decreased well-being during the COVID-19 pandemic [32]. Recent evidence suggests behavioral changes initiated during the COVID-19 pandemic remain today, including a consistent, widespread, and significant decline in physical activity [33]. This study’s findings can inform initiatives and discussions with HPS to identify sustainable and impactful strategies to foster student wellbeing and effectively manage stress.

Finally, the methods employed in this study provide a unique perspective on pharmacy student stress. While this study provided insight into how students were spending their time as well as their perspective on their stress and ways they coped with stress, it is important to note the study’s limitations. First, data were collected from a single institution with a small sample size consisting of student volunteers. This sample size was intentional given the exploratory nature of the study. Additionally, a few participants did not complete all aspects of the study, which limited analyses that could be run at the participant-level. Second, the study focused on students in didactic coursework, as it was hypothesized student time use and stress may vary when compared to the clinical learning environment. Future research should explore the relationship between student time use and stress in the clinical learning environments, as these experiences are an integral part of pharmacy student and HPS training. Further, this study examined a single ‘typical’ course week, which only provides a single snapshot of student stress. Longitudinal studies suggest that stress levels fluctuate from year to year [3, 4]. Due to these limitations, future research is needed to diversify geographical sampling, increase the sample size, and include additional time points during the academic year to enhance generalizability.

## Conclusion

Health profession schools are placing a greater emphasis on graduating well-rounded professionals who are able to take care of patients at the highest possible level. It is critical for students to have mental and physical well-being to care for themselves so they can care for others in the healthcare environment. Some stress can be beneficial for student achievement if it is viewed as a challenge, however, challenges can become hindrances and impede achievement. Overall, increased stress levels among students is a concerning trend that affects students’ mental health and therefore limits their ability to perform to their greatest potential. Better understanding of the relationship between time use and stress is critical to improve the quality of life of students in the health professions.

## Electronic supplementary material

Below is the link to the electronic supplementary material.

Supplementary Material 1

## Data Availability

The full dataset gathered for the study is available from the corresponding author on reasonable request.

## List of Abbreviations

ADL: Activities of daily living
APPE: Advanced pharmacy practice experience
HPS: Health professions students
OSCE: Objective Structured Clinical Examination
PharmD: Doctorate of Pharmacy
PY: Pharmacy Year
PSS10: 10 item Perceived Stress Scale
