# Supplementary material for: There’s no time for no stress! Exploring the relationship between pharmacy student stress and time use
Source: BMC Med Educ. 2023 Apr 24;23:279. doi: 10.1186/s12909-023-04266-5 (PMC10124683; doi:10.1186/s12909-023-04266-5)
Supplement: Supplementary file 1 — Supplementary Material 1 [file 12909_2023_4266_MOESM1_ESM.docx]

**Appendix A**. Semi-structured Focus Group Script

*Think about your time logging week, which involved the baseline stress assessment, time log, daily stress assessment, and final stress assessment….*

1. Thinking about your time logging week, how would you describe the relationship between time use and stress in your life?
   1. Was there anything that surprised you about your time-logging?
2. Reflecting on the causes of your stress and how you alleviate stress on a daily basis, talk to me about how effective your current methods for coping with stress are. Explain. Please provide examples.
3. Reflecting on the causes of your stress and how you alleviate stress on a daily basis, talk to me about how ineffective your current methods for coping with stress are. Explain. Please provide examples.

*Now we want to ask you about specific ways in which you use your time and how that influences your stress.*

1. Talk to me about how your academic time use relates to your stress?
2. Talk to me about how your personal and social time use relates to your stress?
3. Talk to me about how your co-curricular time use relates to your stress?
4. Talk to me about how your time spent working for pay relates to your stress?
5. What else do you see contributing to your stress that we have not yet discussed?
